# Directional Effects of Self-Regulation and Self-Efficacy Changes Within a Weight-Loss Treatment Focused on Exercise and Sweets Consumption: Accounting for Emotional Eating in Women with Obesity

**DOI:** 10.3390/nu17193048

**Published:** 2025-09-24

**Authors:** James J. Annesi

**Affiliations:** Kinesiology Department, School of Health Sciences and Human Services, California State University, Monterey Bay, Seaside, CA 93955, USA; jamesannesi@gmail.com; Tel.: +1-404-579-1809

**Keywords:** obesity, cognitive-behavioral, self-regulation, self-efficacy, exercise, sweets, emotional eating

## Abstract

**Background/Objectives:** Emotional eating is an important factor in the behavioral treatment of obesity, especially in women. Improvements in both exercise frequency and sweets intake have demonstrated positive changes in weight for this subgroup. However, the psychosocial mechanisms of those factors are minimally understood, and any favorable results have largely been transient. Within cognitive-behavioral treatments, increasing self-regulation and self-efficacy have been intervention targets, however, more data on their temporal effects, interrelationships, and specific foci are required to improve weight-loss outcomes. **Methods:** Women with obesity and either high emotional eating (*n* = 54) or low emotional eating (*n* = 52) levels participated in a 6-month cognitive-behavioral treatment. **Results:** Two models were specified: (a) where change in self-regulation predicted weight losses over six and twelve months mediated by changes in self-efficacy leading to behavioral changes; and (b) where self-efficacy was instead the predictor variable, followed by self-regulation changes. Two significant paths of improvement were observed: (a) a merged measure of self-regulation → eating self-efficacy → sweets intake → weight, and (b) a merged measure of self-efficacy → exercise self-regulation → weight. Predictive strengths were generally unaffected by emotional eating level (high or low). Together, improvements in sweets intake and exercise, but not fruit/vegetable consumption, significantly accounted for weight loss for both groups. **Conclusions:** The findings suggest that behavioral weight-loss treatments focus first on self-regulation, then on self-efficacy, and target sweets intake and exercise in women with obesity, independent of their emotional eating levels.

## 1. Introduction

In the United States (U.S.), obesity (i.e., a body mass index [BMI] of at least 30 kg/m^2^) continues to rise, and presently impacts 46% of women in their mid-twenties or older [1]. The decades-long upward trend in obesity is projected to escalate to ~60% by 2050 [1]. That high prevalence is associated with increases in medical disorders including type 2 diabetes mellitus; various forms of cancer; heart, kidney and liver disease; sleep apnea; and depression [2]. Emotional eating is a key, and possibly the strongest, psychological correlate of obesity [3]. This is especially true of women, whose mental health tends to be more closely linked to weight and weight-management issues than men [4]. Since increased exercise and healthier/reduced-energy eating is extremely difficult to maintain via behavioral approaches [5,6], invasive medical methods such as bariatric surgery and an ongoing use of pharmaceuticals are increasingly relied upon to counter excess weight [7,8]. These medical interventions can be expensive and pose health risks of their own.

For those with obesity and also high emotional eating, reducing their intake of sweets has been a constructive treatment target [9]. A focus on increasing fruits and vegetables has also been effective for lowering weight via its association with an overall healthy diet [10,11]. Exercise is independently associated with weight loss; however, adherence is problematic with dropout rates ranging from 50–60% within the first several months of initiating a program [12,13]. Governmental suggestions indicate that a minimum of 150 minutes per week of moderate or greater-intensity exercise, and possibly more than 250 minutes per week, is required for meaningful weight loss [14]. This might be an unreasonable aim considering that less than 4% of American adults of all weights complete even the more conservative amount of 150 minutes per week [15]. However, other research suggests that lower and less-aversive exercise regimens might be easier to maintain [16] and are also consistent with weight loss in adults with obesity [17]. Psychological improvements associated with just moderate increases in exercise [18,19,20,21] affect changes in eating (where the greatest kilocalorie [kcal] deficits are possible [22]).

Although largely atheoretical and limited in effect, education on healthy eating and exercise has been the predominant basis of weight-loss treatments for decades [5,6]. This flawed reliance on the provision of such information as a means to attain and sustain behavioral improvements has been slow to change [23]. However, some researchers suggest that an enhanced understanding of psychosocial correlates of weight loss, and the leveraging of evidence-based behavior-change theories to improve exercise and eating behaviors, will be required to amend the status quo [24,25]. If successful, the treatment processes associated with this approach could either counter current dependencies on surgery and medications or, at minimum, serve as a valuable adjunct to them. Emanating from social cognitive theory [26], self-regulation theory [27], and self-efficacy theory [28]—and their foci on environmental, psychological, and behavioral factors—some behavioral weight-loss treatment research has centered on increasing participants’ self-regulatory (S-Reg) skills (e.g., internal talk, relapse prevention, goal setting, and progress monitoring) and their self-efficacy (S-Eff; perceived ability) around increasing exercise and improving eating behaviors [21,29,30,31]. Consistent with coaction theory [32], the generalization of S-Reg and S-Eff to exercise and eating behaviors has also been suggested [21,29,30,31]. These theories collectively highlight the potential for individuals to gain greater control over impediments to improving their health behaviors.

While obesity treatment studies focused on S-Reg and S-Eff have generally been productive, important gaps in the related research remain [29,33,34,35]. Addressing those gaps are crucial for evaluating their longer-term effects on weight loss, as much of the associated research has relied on cross-sectional data. Such investigations have also often terminated prior to the expected regains in weight, which typically occur around six months after program initiation [5,6]. S-Reg and S-Eff have most often been addressed simultaneously as predictors, meaning that the temporal effects of one on the other could not be determined. For example, it is possible that early changes in S-Reg could predict subsequent changes in S-Eff, which may subsequently lead to changes in exercise, eating, and weight loss. It is feasible that improved S-Reg skills usage may foster increased S-Eff through an enhanced feeling of control over such challenging behavioral changes [27]. However, early changes in S-Eff may instead predict subsequent increases in S-Reg, behavioral, and weight improvements, as increased confidence leads to greater behavioral effort [26,28]. It is also plausible that such paths differ when predicting changes in exercise vs. eating, and/or in consideration of changes prior to expected weight regains (i.e., baseline–Month 6) vs. beyond that time frame (e.g., baseline–Month 12). Resolution of each of those queries could inform treatment designs and contents for both greater reliability and enhanced outcomes.

Although most of the existing research presents the effects of behavioral treatment (possibly theory-based) on weight outcomes, only a small minority of studies also address theory-driven psychosocial mediators (e.g., S-Reg, mood, body image, or S-Eff). Even fewer investigations have attended to theoretically centered interventions’ separate effects on exercise and eating via longitudinal, path analytic frameworks. Such an investigative paradigm could inform where and when intervention foci are most productive for both short and longer-term weight losses [24,25]. Because emotional eating might affect such findings, accounting for participants’ emotional eating propensities could be additionally constructive.

Thus, this field-based investigation focused on women with obesity, while accounting for effects emanating from their degree of emotional eating. To enhance both the applicability and generalization of findings, a community-based setting was incorporated. The present cognitive-behavioral treatment focused on increasing participants’ S-Reg and S-Eff, as applied to both exercise and eating, while assessing their effects on weight. Because of its relevance to both emotional eating specifically [9], and weight-reduction generally [36], sweets intake was the eating behavior specified within the analyses of paths toward weight changes after six and twelve months. It was hoped that findings extend both the existing theory and effectiveness of behavioral obesity treatments through an improved understanding of temporal effects of relevant psychosocial variables. This study’s hypotheses and research questions were as follows:

- There will be significant improvements in S-Reg, S-Eff, sweets intake, fruit/vegetable consumption, exercise, and weight. It was addressed as a research question whether those improvements will significantly differ by emotional eating group (high or low).
- Another research question was whether paths from early changes in S-Reg leading to (→) longer-term changes in S-Eff → behavioral (i.e., exercise and sweets intake) changes → weight losses will be significant, and/or whether similar paths initiating from S-Eff → S-Reg changes will be significant.
- A final research question was whether the above paths would be significantly affected by emotional eating level.
- It was expected that a reduction of sweets intake would account for a greater portion of the explained variance in weight loss over six and twelve months than an increase of fruit/vegetable consumption and/or exercise.

## 2. Materials and Methods

### 2.1. Participants

Participant data were collected from an ongoing course of field-based research in the U.S. primarily focused on contrasting differing behavioral means for weight-loss within community settings. Based on sample size requirements given in the *Data analyses* subsection below, the most recent participants recruited via local newspapers and social media and fulfilling the present inclusion criteria prior to COVID-19 restrictions, were included. Those criteria were: (a) being a woman of ≥21 years with obesity, (b) no current/soon planned pregnancy or physical contraindication for safe participation, and (c) no weight-management program participation or change in psychotropic medication (including dosage) within the previous 12 months. There was no cost or compensation for volunteering. Based on a median split of baseline Emotional Eating Scale scores (see the *Measures* subsection below [37]), the included participants were divided, post hoc, into groups of high emotional eating (*n* = 54) and low emotional eating (*n* = 52). Based on that criterion, those in the high emotional eating group had mean item responses indicating a moderate-strong propensity to eat associated with negative affect. Considering values indicated within previous research [37,38,39,40], that pattern of responses was also consistent with the highest 80th percentile for emotional eating among all adults. There was no significant difference observed, based on any assessed demographic variable, by emotional eating group. The overall mean age was 46.6 years (*SD* = 9.6); mean BMI was 34.5 kg/m^2^ (*SD* = 3.2); racial/ethnic makeup was 72% White, 20% Black, 7% Hispanic, 1% other; and the educational makeup was 73% bachelor’s degree or greater and 27% high school or some college. All but two participants self-reported within the middle family-income range of US$50,000–US$150,000/year. Ethical and privacy mandates of the World Medical Association’s Declaration of Helsinki and the American Psychological Association were maintained throughout. A university institutional review board approved the study protocol and the required signed informed consent was attained from each participant.

### 2.2. Measures

#### 2.2.1. Self-Regulation

The Exercise-Related Self-Regulation Scale (sample item, “When I get off-track with my exercise plans, I work to quickly get back to my routine”) [41] and the Eating-Related Self-Regulation Scale (sample item, “I say positive things to myself about eating well”) [41] each had 10 items indicating the extent of usage of S-Reg skills applied to exercise or eating, respectively. The skills embedded within items were previously defined within research reviews as being consistent with social cognitive theory and/or operant conditioning theory [42,43]. Response options for the respondent’s S-Reg skills usage “currently” ranged from 1 (*never*) to 4 (*often*), and a mean item score was calculated for each scale. A higher score indicated greater S-Reg skills usage. Internal consistencies were reported to be Cronbach’s α = 0.79 and 0.81, respectively (present sample, αs = 0.75 and 0.75, respectively), and 2-week test–retest reliabilities were 0.78 and 0.74, respectively [41]. An aggregate scale of exercise- and eating-related S-Reg (i.e., S-Reg-merged) was scored as the mean of item responses, weighted for the number of items in each scale (possible range = 1–4).

#### 2.2.2. Self-Efficacy

To assess the degree of confidence in persisting with exercise and healthy eating under challenging conditions (i.e., exercise S-Eff and eating S-Eff, respectively), the 5 items of the Exercise Self-Efficacy Scale [44] (sample item, “I am tired”) and the 20 items of the Weight Efficacy Life-Style Questionnaire [45] (sample item, “I can resist eating when I am depressed [or feeling down]”) were incorporated. After a minor modification to obtain symmetry across the scale scoring formats, response options ranged from 1 (*not confident*) to 10 (*very confident*) for both, and a mean item score was calculated for each scale. A higher score indicated greater S-Eff. For the Exercise Self-Efficacy Scale, internal consistencies were reported to be Cronbach’s α = 0.76–0.82 (present sample, α = 0.74), and 2-week test–retest reliabilities ranged from 0.74–0.78 [44]. For the Weight Efficacy Life-Style Questionnaire, internal consistencies were reported to range from Cronbach’s α = 0.70–0.90 [45] (present sample, α = 0.77). An aggregate scale of exercise- and eating-related S-Eff (i.e., S-Eff-merged) was scored as the mean of item responses from both scales, weighted for the number of items in each scale (possible range = 1–10).

#### 2.2.3. Exercise

The Godin–Shephard Leisure-Time Physical Activity Questionnaire [46] was used to measure exercise level (also physical activity level, as defined as a less structured form of exercise). The number of ≥15-min bouts of “mild intensity” (e.g., normal-paced walking), “moderate intensity” (e.g., fast-paced walking), and “strenuous intensity” (e.g., running) exercise during the previous 7 days were recalled. Each of those activities was then assigned a corresponding score of 3, 5, or 9 metabolic equivalents (METs; a measure of energy expenditure beyond the resting state), respectively. After multiplying the number of bouts × MET score for each, the results were summed. The Godin–Shephard Leisure-Time Physical Activity Questionnaire MET scores demonstrated robust validity through their associations with accelerometry, body fat, and VO_2_ max treadmill test results, and the 2-week test–retest reliability was 0.74 [47,48,49,50,51]. This measure has been incorporated regularly in medical research [52].

#### 2.2.4. Eating Behaviors

A previously applied food frequency recall instrument [11] measured sweets intake and fruit/vegetable consumption “during a typical day over the previous 7 days.” For sweets, respondent-reported portions of consumed sweets (e.g., a medium-size [59 mL] cookie; a 355 mL sugar-sweetened beverage) were summed. For fruits/vegetables, respondent-reported (combined) portions of fruits (e.g., small apple [118 mL if canned]) and vegetables (e.g., 118 mL green beans) consumed were summed. Portion sizes and corresponding instructions were based on U.S. governmental sources [53,54]. Those instructions also accounted for mixed foods (e.g., salads) and large and small portion sizes. They also directed respondents to exclude fried vegetables/fruits, and if a fruit was combined with a sweet (e.g., candy-coated apple), it was to be counted as a sweet. The 3-week test–retest reliabilities in women with obesity were reported to range from 0.77–0.83, and concurrent validity was indicated through correspondences with strongly validated, but lengthier, food recall surveys (e.g., the Block Food Frequency Questionnaire) [55,56].

#### 2.2.5. Emotional Eating Propensity

Propensity to engage in emotional eating when prompted by a challenging emotional state was measured using 15 items (sample items, “sad,” “frustrated”) of the Emotional Eating Scale [37]. Response options ranged from 0 (*no desire to eat*) to 9 (*an overwhelming urge to eat*), and were summed. A higher score indicated more emotional eating. Internal consistencies were reported to range from Cronbach α = 0.72–0.79 (study sample, α = 0.74), and the 3-week test–retest reliability was 0.79 [37,57].

#### 2.2.6. Body Composition

Body weight was measured to the nearest 0.10 kg using a self-zeroing digital floor scale (Health o meter® Model 800KL; McCook, IL, USA) calibrated the day of each assessment. Heavy outer clothing and shoes were removed by each participant prior to measurement. To confirm the inclusion criterion of having obesity (BMI ≥ 30 kg/m^2^), each participant’s height was measured to the nearest 0.10 cm using a stadiometer (Health o meter® PORTROD; McCook, IL, USA), enabling calculation of BMI. Non-instructional study staff completed the weight and height measurements.

### 2.3. Procedure

Treatment instructors were currently employed staff members of the participating community centers who also desired to serve as an instructor within the present research. Each had at least one national/international certification that was health related. They were trained by study staff in the present weight-management protocol. That treatment required a total of 10–11 h of participant time. Its contents, adapted from National Institutes of Health/National Cancer Institute-certified obesity programming, were based on key aspects of social cognitive theory [26], self-regulation theory [27], and self-efficacy theory [28]. Each of those theories emphasize the potential for individuals’ goals toward weight loss to progress via increased exercise and dietary improvement by addressing interrelations between environmental, psychological, and behavioral factors. Thus, toward those aims, the present program emphasized increasing participants’ S-Reg skills usage and their S-Eff.

There was a combination of individual and small-group instruction over six months, generally meeting every two weeks in groups after several weekly one-on-one sessions were completed. Each session was 50 min in length. Increased S-Reg skills development was sought through coaching, rehearsal, and providing brief hand-outs on theory-driven techniques [42] that included goal setting, progress tracking, stimulus control, cognitive restructuring, attentional control, and dissociation from discomfort. Increases in S-Eff were pursued through the four suggested self-efficacy theory-based means [58]: (a) “mastery experience” (e.g., through a participant’s emphasis on S-Reg methods leading to their increased feelings of control over challenges and barriers); (b) “social persuasion” (e.g., through instructor-initiated verbal praise/reinforcement to each participant); (c) “vicarious reinforcement” (e.g., through instructor-supplied illustrations of successes from individuals similar to the participants); and (d) “emotional/psychological states” (e.g., through identification of exercise-associated mood improvements to the participant). As articulated within coaction theory [32], generalization/carry-over of both S-Reg methods and S-Eff across exercise and eating behavior contexts was sought throughout. The primary behavioral foci of the treatment were on increasing exercise using self-selected modalities (walking, in most cases), increasing fruit/vegetable consumption, reducing intakes of sweets, and maintaining a daily energy intake of 1200–1500 kcal.

Although basic education on weight-loss methods and the psychological aspects of eating was provided throughout the treatment, approximately 90% of instructor–participant contact time was related to advancements in S-Reg and S-Eff. Participants were directed to the U.S. governmental website of www.myplate.gov for additional nutrition education (based on the degree desired by each participant). The ≥150 min/week of moderate or greater intensity exercise for health benefits [59] was mentioned, but not emphasized. Because of its positive effects on weight loss previously indicated [60], weekly self-weighing was encouraged. However, it was not reported as data. Structured fidelity checks were completed by direct observation of 10% of treatment sessions by non-instructional study staff. Those reviews indicated strong protocol compliance requiring only minor modifications to be carried out by some instructors. The same study staff conducting fidelity appraisals administered study measures to participants in a private area, where the corresponding data were kept private.

### 2.4. Data Analyses

Based on preconditions indicated by White et al. [61], there was no systematic bias in participants with vs. without missing data. Thus, the corresponding missing-at-random categorization supported use of the expectation–maximization (EM) algorithm for imputation [62,63]. For the primary regression analysis, a sample size of ≥91 was required to identify a moderate effect of *f*^2^ = 0.15 at the statistical power of 0.80, α < 0.05 [64].

In response to the first hypothesis/research question, and based on suggested processes [65], mixed repeated-measures analyses of variance (ANOVA) first assessed overall gains (changes) from baseline on the measures of S-Reg, S-Eff, exercise, sweets and fruit/vegetable intake, and weight; then whether those change terms significantly differed between the high and low emotional eating groups. Effect sizes were calculated using partial eta-squared (η^2^_partial_), where values of ~0.01, ~0.06, and ≥0.14 are considered small, moderate, and large effects, respectively. In addressing the next research questions, data aggregated across the participant groups was used to conduct two families of path analyses. In what is being referred to as Model 1, a 3-month change from baseline on the merged (exercise and eating) measure of S-Reg predicted weight change over both 6 and 12 months, serially mediated by 6-months changes in S-Eff, then behavior. One path within that model incorporated exercise-related S-Eff, then exercise, while the other path integrated eating-related S-Eff, then sweets intake. In analyses following up from Model 1 results, high and low emotional eating (coded 1 and 0, respectively) were entered as a covariate. Model 2, instead, initiated from change in the merged measure of S-Eff, and S-Reg applied to exercise and eating replaced the corresponding S-Eff measures. The other aspects were consistent with Model 1. Finally, in response to the final hypothesis, sensitivity analyses applied multiple linear regression to evaluate the proportion of variance in 6- and 12-month weight changes independently accounted for by 6-month change in sweets intake along with changes in exercise and/or fruit and vegetable consumption, also over 6 months.

Statistical significance was set at α < 0.05 (two-tailed). Where bootstrapping was applied, a 95% confidence interval (95% CI) assessed significance. Statistical analyses were carried out using SPSS Statistics Version 28.0.1.0 (IBM, Armonk, NY, USA), incorporating the PROCESS Version 4.2 macroinstruction Model 82 with 10,000 percentile-based bootstrapped re-samples [66]. That PROCESS model enabled separate analyses of paths from baseline–Month 3 changes in S-Reg-merged and S-Eff-merged toward weight changes over 6 and 12 months, while simultaneously assessing their combined predictive strength. Reporting followed TREND guidelines [67].

## 3. Results

### 3.1. Changes in Study Variable Scores

Descriptive statistics for study variables and the results of mixed repeated-measures ANOVAs are presented in Table 1. There were significant overall improvements across all variables (*p*s < 0.001, η^2^_partial_-values = 0.34–0.71). Emotional eating group × time differences were significant for only sweets intake, S-Eff-merged, and eating S-Eff. In those cases, positive changes were significantly greater in the high emotional eating group.

### 3.2. Regression and Path Analyses

**Figure 1:**
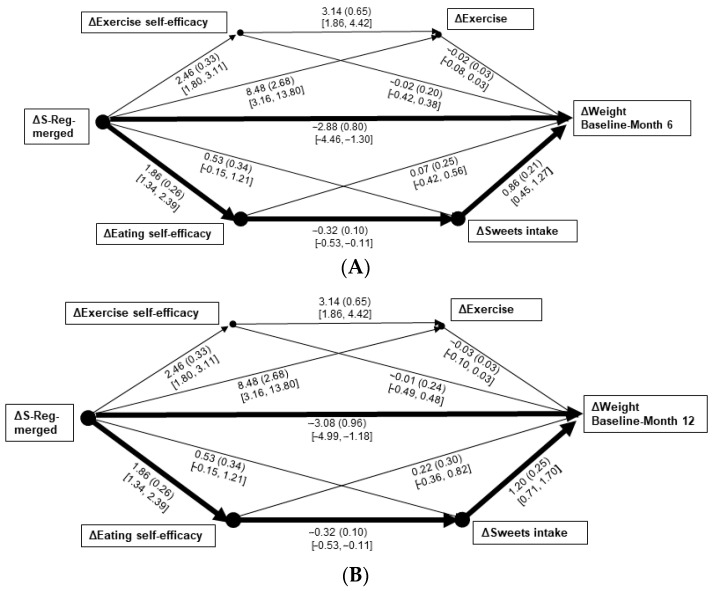
(**A**,**B**) Mediation paths of 3-month changes in the merged measure of self-regulation to weight changes from baseline–Month 6 (**A**) and baseline–Month 12 (**B**), through changes in self-efficacy, then changes in the assessed behaviors of exercise and sweets intake. Δ = change in measure score. Individual bootstrapped path data are given as B (*SE*_B_) [95% confidence interval]. A heavy line indicates a significant path from self-regulation change toward weight change.

Intercorrelations of study variables are presented in Table 2. Model 1 significantly predicted changes in weight from baseline–Month 6, *R*^2^ = 0.35, *F*(5, 100) = 10.87 and baseline–Month 12, *R*^2^ = 0.34, *F*(5, 100) = 10.19, *p*s < 0.001. Only the paths from change in S-Reg-merged → S-Eff-eating change → sweets intake change → weight changes were significant: B = −0.51, *SE*_B_ = 0.18, 95% CI [−0.93, −0.17] (Figure 1A) and B = −0.72, *SE*_B_ = 0.28, 95% CI [−1.37, −0.26] (Figure 1B), respectively. When the emotional eating group (high or low) was subsequently added to those models as a covariate, the overall prediction strengths of the weight changes were similar to when it was left unaccounted (*R*^2^s = 0.37 and 0.34, respectively). The significant path findings remained unchanged. However, group significantly strengthened predictions of changes in S-Eff-eating, B = 1.00, *SE*_B_ = 0.26, 95% CI [0.48, 1.52], and sweets intake, B = −0.86, *SE*_B_ = 0.31, 95% CI [−1.47, −0.25].

**Figure 2:**
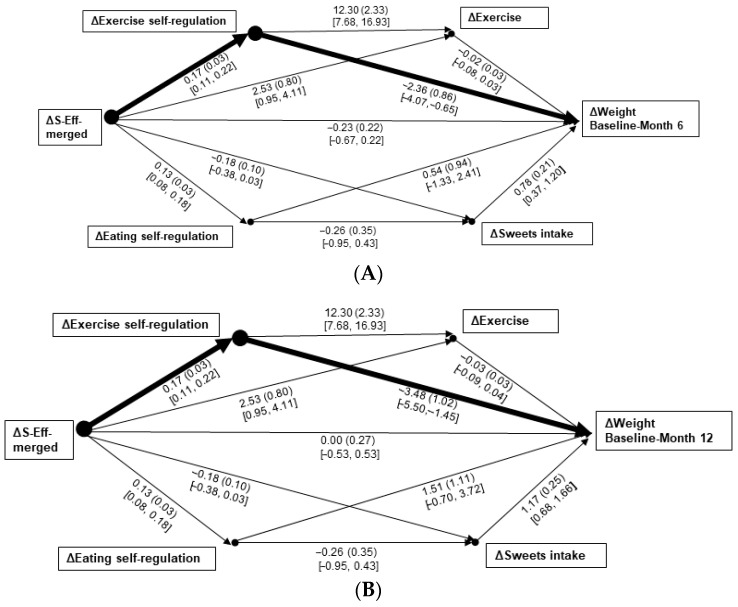
(**A**,**B**) Mediation paths of 3-month changes in the merged measure of self-efficacy to weight changes from baseline–Month 6 (**A**) and baseline–Month 12 (**B**), through changes in self-regulation, then changes in the assessed behaviors of exercise and sweets intake. Δ = change in measure score. Individual bootstrapped path data are given as B (*SE*_B_) [95% confidence interval]. A heavy line indicates a significant path from self-regulation change toward weight change.

Model 2 significantly predicted changes in weight from baseline–Month 6, *R*^2^ = 0.33, *F*(5, 100) = 9.90, and baseline–Month 12, *R*^2^ = 0.35, *F*(5, 100) = 10.55, *p*s < 0.001. Only the paths from change in S-Eff-merged change → exercise change → weight changes were significant: B = −0.39, *SE*_B_ = 0.19, 95% CI [−0.82, −0.09] (Figure 2A) and B = −0.58, *SE*_B_ = 0.25, 95% CI [−1.13, −0.19] (Figure 2B), respectively. When emotional eating group was subsequently added to those models as a covariate, the overall prediction strengths of the weight changes were similar to when it was unaccounted (*R*^2^s = 0.36 and 0.35, respectively). The significant path results remained unchanged. However, group significantly strengthened predictions of changes in sweets intake, B = −1.02, *SE*_B_ = 0.30, 95% CI [−1.62, −0.43].

### 3.3. Sensitivity Analyses: Prediction of Weight Changes via Behavioral Changes

Reductions in weight over 6 and 12 months (−6.1% and −5.8%, respectively) were significantly predicted by simultaneous entry of 6-month changes in sweets intake and exercise, *R*^2^_adjusted_ = 0.23 and 0.25, respectively (*p*s < 0.001). Both changes in sweets, B = 0.72, *SE*_B_ = 0.21 and B = 1.04, *SE*_B_ = 0.25, respectively (*p*s < 0.001) and exercise, B = −0.08, *SE*_B_ = 0.02, *p* < 0.001 and B = −0.08, *SE*_B_ = 0.03, respectively (*p* = 0.002) independently contributed to the explained variances in weight change. Those weight reductions were also significantly predicted by simultaneous entry of 6-month changes in sweets and fruit/vegetable intake, *R*^2^_adjusted_ = 0.18 and 0.23, respectively (*p*s < 0.001). However, only changes in sweets intake independently contributed to the explained variance, B = 1.22, *SE*_B_ = 0.26, and B = 0.90, *SE*_B_ = 0.22 (*p*s < 0.001).

When changes in sweets intake, exercise, and fruit/vegetable consumption were simultaneously entered as predictors, weight changes were, again, significantly predicted, *R*^2^_adjusted_ = 0.23 and 0.24, respectively (*p*s < 0.001). Only changes in sweets intake, B = 0.74, *SE*_B_ = 0.2, and B = 1.06, *SE*_B_ = 0.25 (*p*s < 0.001) and exercise, B = −0.09, *SE*_B_ = 0.02 (*p* < 0.001) and B = −0.09, *SE*_B_ = 0.03 (*p* = 0.002), respectively, independently contributed to the explained variance. When emotional eating group was entered into Step 2 in each of the above equations, it did not significantly increase the explained variance in weight change over either 6 months (*p*s > 0.20) or 12 months (*p*s > 0.70).

### 3.4. Post Hoc Analyses

Post hoc probative analyses indicated that baseline scores for the high emotional eating group were significantly higher on sweets intake, *t*(104) = 3.17, *p* = 0.002, *d* = 0.61, and lower on eating S-Eff, *t*(104) = 4.55, *p* < 0.001, *d* = 0.88, and S-Eff-merged, *t*(104) = 2.65, *p* = 0.008, *d* = 0.51. For those variables, the baseline scores were significantly (inversely) associated with the change terms, βs = −0.88, −0.68, and −0.52, respectively (*p*s < 0.001).

## 4. Discussion

Consistent with the present aims, social cognitive theory was leveraged to advance the understanding of psychosocial changes’ impact on weight-loss behaviors. The research also facilitated an improved comprehension of how obesity treatments might be better shaped. That included accounting for emotional eating. In concurrence with the initial hypothesis, the cognitive-behavioral treatment, which emphasized improvements in S-Reg and S-Eff over traditional education in nutrition and exercise methods, was associated with significant improvements in those psychosocial variables, as well as in sweets intake, fruit/vegetable consumption, and weight loss. Effect sizes for weight losses were large, with the observed reductions in weight largely enabling the minimum 5% loss required to lessen health risks [68].

Improvements in S-Eff variables and sweet intake was significantly greater in the high emotional eating group. However, their baseline scores were also less favorable initially, which was likely a reason for their greater improvements. Also in support of the corresponding hypothesis, changes in the targeted behavior of sweets intake, but not fruit/vegetable consumption, explained a significant independent portion of the variance in weight loss. Increased exercise also uniquely accounted for a significant portion of the explained variance in weight loss. However, when paired with changes in sweets intake, its prediction strength was somewhat weaker. Thus, for the current type of participant seeking lost weight (i.e., women with obesity), the present focus on fostering reductions in sweets intake along with increased exercise was reinforced. Those findings are consistent with some research [36,69], but not others, where large amounts of exercise and specific types of diets with a plethora of nutritional requirements are advocated for weight reduction [14,70,71]. Improvements in sweets intake and exercise also appear to be an effective treatment focus for women with both lower and higher propensities for emotional eating.

The research questions related to the temporal and directional effects of changes in S-Reg and S-Eff—and their interrelations within separate paths toward the changes in the behavioral variables and weight—also yielded findings of considerable value. Specifically, the only significant paths including both S-Reg and S-Eff indicated that early change in overall S-Reg leads to eating-related S-Eff over a longer-term, sweets reduction, and weight losses over both 6- and 12-month periods (see Figure 1A,B). That finding suggests that an initial focus on fostering S-Reg skills, that then extends toward increased feelings of control over eating (manifesting as a reduction in sweets intake), is a viable path toward weight reduction; whereas the significant paths from early change in overall S-Eff to exercise-related S-Reg and directly to weight losses (see Figure 2A,B) suggests a different pattern regarding increased exercise. It should be noted; however, that bivariate relations between changes in exercise and weight was significant at a similar strength to that of change in sweets intake (see Table 2). Thus, for exercise, an initial treatment focus on bolstering S-Eff appears productive through its impact on increasing exercise-related S-Reg.

Consistent with the theory background [26,27,28] and other related research findings [18,29,31,34,35], S-Reg and S-Eff were confirmed as important intervention targets. Also in agreement with such previous analyses, an initial focus on eating-related S-Reg and an emphasis on S-Eff toward exercise appeared to be quite viable within behavioral weight-loss treatments [21]. One possibility for such differences in directionality might be that initial feelings of ability are paramount given the high likeliness for early exercise drop out [12]. Thus, an increased perception of ability (i.e., S-Eff) might be key to enable enough of a commitment to fully apply newly learned S-Reg skills concerning maintained exercise. However, with changes in eating, patterns of unhealthy, high-kcal eating (typical across the U.S. population [72]) might require early and tenacious applications of S-Reg skills. Those can then facilitate enhanced feelings of competence and sustained behavioral changes. This could be especially relevant where those patterns have persisted for periods long enough to sustain an individual’s excess weight.

It is beyond the scope of this research to determine how the demands of *increasing* exercise and *decreasing* existing unhealthy eating patterns contrast in regard to the directionalities of the assessed psychosocial factors. However, the ability to assess paths toward weight loss through both eating and exercise changes separately, but also via aggregating their effects, was a strength of this research. This is important because changes in both behaviors are essential for sustained reductions in weight [73]. Although the findings enable theory-driven advances that are generalizable to practice, there were also limitations to this study. Those limitations include: (a) a sample of primarily white women with obesity of the middle socioeconomic stratum of the U.S. whose volunteerism could suggest high motivation; (b) absence of a control/comparison condition that could reduce expectation and/or social support biases; (c) a somewhat arbitrary cutoff point for (high/low) degree of emotional eating (especially because individuals with obesity tend to be high in emotional eating; [74]); and (d) reliance on self-reported measurements. To minimize the effects of volunteerism, treatment enrolments enacted by medical professionals could help with the inclusion of those in need, but with minimal initial motivation. Replications and extensions with individuals with severe obesity (exclusively), diabetes, and/or mental challenges, along with those undergoing medical weight-loss methodologies, will also be important. Additionally, while the selection of S-Reg and S-Eff were well justified, other theory-based psychosocial factors (e.g., anxiety, locus of control, body image, depression) could also yield productive findings. Even given these limitations, it is hoped that the present findings will be integrated into further research and practice to improve the lagging results of the typical educationally based obesity treatment formats [5,6,23]. Ultimately, physiological pathologies and reductions in quality-of-life associated with obesity [2,75] will be improved through the incorporation of evidence-informed behavioral processes.

## Figures and Tables

**Table 1:** Descriptive statistics of study variables and results of group × time contrasts.

	Baseline		Month 3		Month 6		Month 12		Group × Time Contrasts		
Grouping	*M*	*SD*	*M*	*SD*	*M*	*SD*	*M*	*SD*	*F*(1, 104)	*p*	η^2^_partial_
Self-regulation-merged											
Low emotional eating	1.90	0.42	2.57	0.29							
High emotional eating	1.81	0.43	2.52	0.36							
Aggregated data	1.86	0.43	2.55	0.32					0.18	0.672	0.00
Self-efficacy-merged											
Low emotional eating	5.16	1.28	6.15	1.19							
High emotional eating	4.41	1.61	6.19	1.64							
**Aggregated data**	**4.78**	**1.50**	**6.17**	**1.56**					**6.15**	**0.015**	**0.06**
Exercise self-efficacy											
Low emotional eating	5.28	1.69			6.71	2.05					
High emotional eating	5.01	2.00			7.01	2.09					
Aggregated data	5.14	1.85			6.86	2.06			1.73	0.191	0.02
Exercise self-regulation											
Low emotional eating	1.90	0.48			2.68	0.25					
High emotional eating	1.72	0.51			2.60	0.41					
Aggregated data	1.81	0.50			2.64	0.34			0.82	0.367	0.01
Eating self-efficacy											
Low emotional eating	5.04	1.24			6.35	1.46					
High emotional eating	3.80	1.55			6.19	1.19					
**Aggregated data**	**4.41**	**1.53**			**6.27**	**1.33**			**11.30**	**0.001**	**0.10**
Eating self-regulation											
Low emotional eating	1.90	0.45			2.60	0.28					
High emotional eating	1.91	0.43			2.58	0.35					
Aggregated data	1.90	0.44			2.59	0.32			0.09	0.764	0.00
Exercise (METs/week)											
Low emotional eating	10.60	6.71			33.66	14.58					
High emotional eating	6.52	7.26			31.91	16.49					
Aggregated data	8.52	7.26			32.77	15.53			0.59	0.443	0.01
Sweets (portions/day)											
Low emotional eating	1.63	1.09			1.12	0.81					
High emotional eating	2.65	2.04			1.06	0.82					
**Aggregated data**	**2.15**	**1.71**			**1.08**	**0.81**			**13.68**	**<0.001**	**0.12**
Fruit/vegetable (portions/day)											
Low emotional eating	4.12	2.09			6.09	2.29					
High emotional eating	3.76	1.81			6.57	1.97					
Aggregated data	3.93	1.95			6.33	2.14			3.54	0.063	0.03
Weight (kg)											
Low emotional eating	95.19	11.54			89.50	11.66	90.37	11.51			
High emotional eating	94.55	11.74			88.62	11.78	88.30	12.39			
Aggregated data	94.86	11.59			89.05	11.68	89.32	11.95	2.66	0.106	0.03

Low emotional eating group *n* = 52. High emotional eating group *n* = 54. Aggregated data *N* = 106. MET = metabolic equivalent of task (a measure of energy expenditure associated with exercise). Partial eta-squared (η^2^_partial_) = *SS*_Effect_/(*SS*_Effect_ + *SS*_Error_), where a small effect is ~0.01, a medium effect is ~0.06, and a large effect is ≥ 0.14. Overall changes for time indicated significant improvements (*p*s < 0.001) for each variable. For weight (the only measure assessed across more than two temporal points), the follow-up contrast (LSD method) indicated significant overall reductions during changes from baseline–Month 6 and baseline–Month 12, and no significant difference from Month 6–Month 12. Significant *p*-values for aggregated data are bolded.

**Table 2:** Intercorrelations of study variables (*N* = 106).

Variable	1	2	3	4	5	6	7	8	9	10
1. ΔSelf-regulation-merged, baseline-Month 3	---									
2. ΔSelf-efficacy-merged, baseline-Month 3	0.67 ^†^	---								
3. ΔExercise self-efficacy, baseline-Month 6	0.59 ^†^	0.77 ^†^	---							
4. ΔExercise self-regulation, baseline-Month 6	0.79 ^†^	0.49 ^†^	0.58 ^†^	---						
5. ΔEating self-efficacy, baseline-Month 6	0.57 ^†^	0.73 ^†^	0.62 ^†^	0.50 ^†^	---					
6. ΔEating self-regulation, baseline-Month	0.78 ^†^	0.46 ^†^	0.50 ^†^	0.73 ^†^	0.49 ^†^	---				
7. ΔExercise, baseline-Month 6	0.55 ^†^	0.50 ^†^	0.62 ^†^	0.59 ^†^	0.56 ^†^	0.52 ^†^	---			
8. ΔSweets intake, baseline-Month 6	−0.02	−0.22 *	−0.19 *	−0.07	−0.25 *	−0.17	−0.26 **	---		
9. ΔWeight, baseline-Month 6	−0.45 ^†^	−0.36 ^†^	−0.36 ^†^	−0.43 ^†^	−0.35 ^†^	−0.34 ^†^	−0.40 ^†^	0.39 ^†^	---	
10. ΔWeight, baseline-Month 12	−0.38 ^†^	−0.28 **	−0.31 **	−0.40 ^†^	−0.29 **	−0.27 **	−0.37 ^†^	0.44 ^†^	0.92 ^†^	---

Δ = change in score during the indicated period. * *p* < 0.05. ** *p* < 0.01. ^†^ *p* < 0.001 (two-tailed tests).

## Data Availability

Based on institutional review requirements for participant anonymity and privacy, the data set supporting the findings within this article will be made available only by reasonable request made to the corresponding author.
